# Hijacking the signal: a critical evaluation of quorum sensing inhibitors as a next-generation approach against *Staphylococcus aureus*

**DOI:** 10.1186/s12964-026-02674-w

**Published:** 2026-02-02

**Authors:** Sama S. Eltaher, Zeina Khattab, Gina Walid, Omar Loay, Rana Emad, Clara Hakim, Mohamed Elhadidy

**Affiliations:** 1 Center for Genomics, Helmy Institute for Medical Sciences, Zewail City of Science and Technology, Giza, Egypt; 2 Biomedical Sciences Program, University of Science and Technology, Zewail City of Science and Technology, Giza, Egypt; 3 Department of Bacteriology, Mycology and Immunology, Faculty of Veterinary Medicine, Mansoura University, Mansoura, Egypt

**Keywords:** *Staphylococcus aureus*, Biofilm, Quorum sensing, Quorum-sensing inhibitors, Accessory gene regulator, Antimicrobial resistance

## Abstract

*Staphylococcus aureus* (*S. aureus*) is an opportunistic, Gram-positive pathogen that forms significant clinical challenges due to its multidrug-resistant mechanisms, diverse virulence factors, and robust biofilm-forming capacity. One of the main drivers of antimicrobial resistance (AMR) is the selective pressure exerted by antibiotic use, necessitating alternative therapeutic strategies. Among these, quorum-sensing inhibitors (QSIs) have emerged as promising candidates for disrupting bacterial communication and reducing virulence without compromising bacterial viability. This review focuses on targeting *S. aureus* communication systems, particularly the accessory gene regulator (*agr*) quorum-sensing system. We first provide an overview of biofilm development strategies in *S. aureus*, define bacterial communication networks, and discuss the advantages and limitations of targeting these systems as a strategy for virulence attenuation. We also explore the interplay between regulatory systems within biofilms and how they influence each stage of biofilm maturation. The agr system comprises a network of proteins that can be selectively targeted to disrupt its signaling cascade. Potential intervention points include (1) obstruction of autoinducing peptide (AIP) synthesis, (2) degradation of preformed AIPs, (3) competitive inhibition or modification of the histidine kinase receptor AgrC, and (4) interference with downstream effectors such as AgrA and RNAIII. Given that the agr system primarily operates in the later stages of biofilm development, facilitating biofilm dispersal and upregulating virulence genes, QSIs alone may attenuate virulence yet risk persistent biofilm-associated infections. Accordingly, we emphasize the importance of combining QSIs with biofilm-disrupting or eradicating agents to reduce both biofilm formation and virulence, while minimizing the risk of resistance emergence. Future research should focus on optimizing such combinatorial strategies, evaluating in vivo efficacy, and ensuring safety and minimal off-target effects to facilitate clinical translation of QSIs as viable anti-virulence therapeutics against *S. aureus* infections.

## Background

**Fig. 1:**
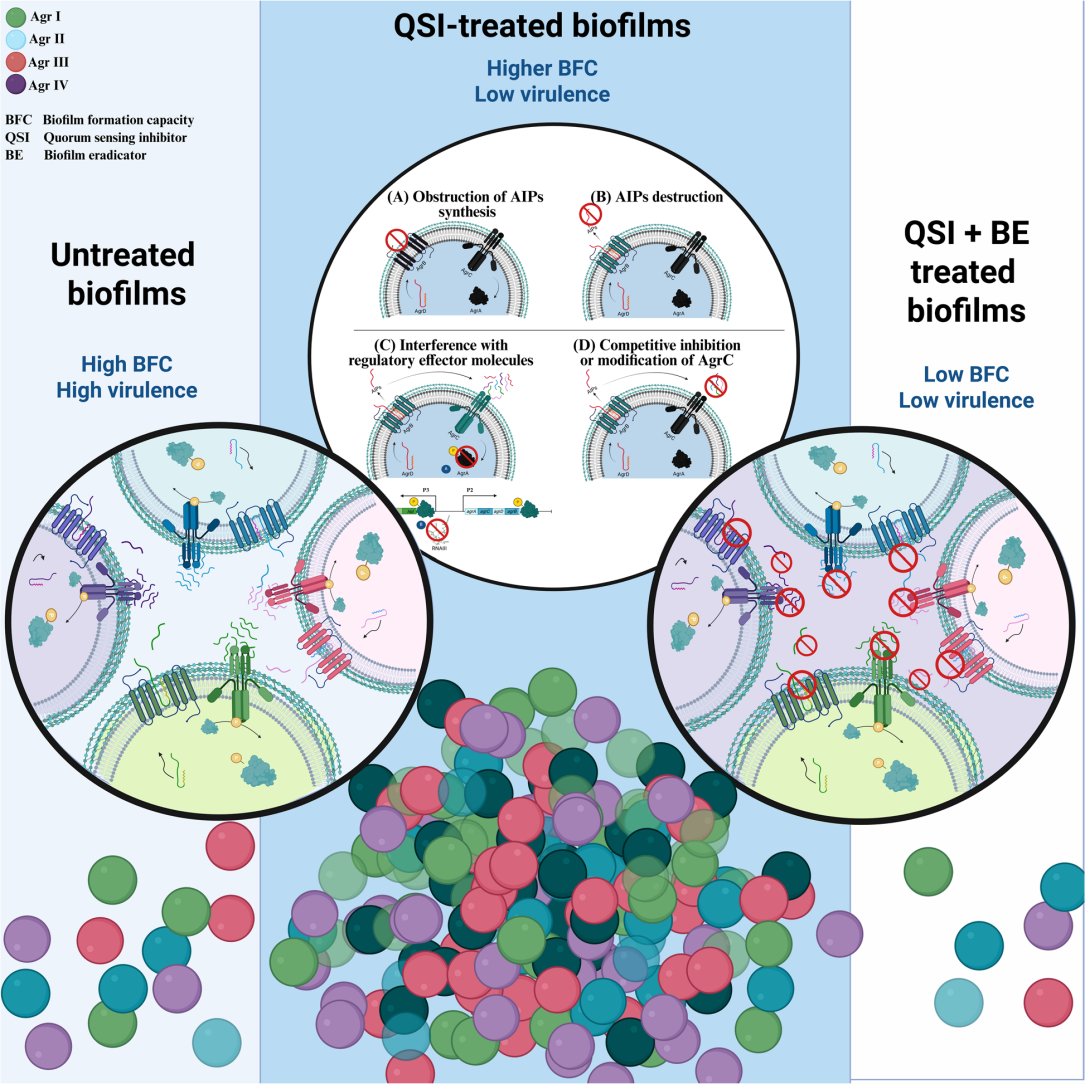
Graphical abstract covering the main conclusion from the review article. *S. aureus* in untreated forms usually exists in virulent states with high biofilm-forming capacities (BFC). Treatment with quorum-sensing inhibitors (QSIs) reduces bacterial virulence but may lead to persistent biofilms; accordingly, it is recommended to use biofilm-eradicators (BE) along with QSIs to synergize beneficial outcomes in terms of virulence factors and biofilm formation potentials. (Created with BioRender.com)

*Staphylococcus aureus* (*S. aureus*) is a Gram-positive pathogen that is a common colonizer of human microbiota, commonly residing in the skin and mucous membranes of healthy individuals [1]. However, it is also considered an opportunistic pathogen capable of causing serious infections, such as endocarditis, bacteremia, and soft tissue infections, upon entry to the bloodstream or internal tissues [2]. Antimicrobial Resistance (AMR) has emerged as one of the most significant healthcare challenges of the twenty-first century [3], underscoring the urgent need for the development of novel alternative therapies [4]. Conventional antibiotics mainly exert selective pressure on bacterial cells through direct bactericidal effects [5], a key driver in the emergence and spread of AMR [3, 6]. Accordingly, there is an urgent need to develop alternative therapies to tackle AMR without mimicking the mechanisms of action of conventional antibiotics. Achieving this goal requires a comprehensive understanding of both the mechanisms of action of the conventional antibiotics and the bacterial strategies underlying AMR (Fig. 1).

Among the most critical contributors to bacterial virulence and AMR is biofilm formation [7, 8]. Bacterial biofilms are organized communities composed of single or multiple bacterial species [9], embedded within a dense mesh-like network of extracellular polymeric substances (EPS) [10]. The protective extracellular matrix (ECM) confers significantly enhanced resistance, with biofilm-associated cells exhibiting resistance levels up to 1,000-fold higher than their planktonic counterparts [11].

This review aims to provide a detailed understanding of biofilm developmental strategies to inform the development of novel therapeutic approaches that mitigate infection severity without promoting further resistance. Particular attention is given to bacterial communication systems, such as quorum sensing, which regulate virulence and biofilm formation. Strategies to disrupt these signaling pathways are also explored. However, these approaches remain controversial, with some evidence suggesting they may lead to unintended consequences more detrimental than the untreated infection itself [12, 13]. Therefore, this review critically evaluates both sides of the debate and proposes balanced, innovative strategies to address the dual challenges of biofilm-associated infections and AMR.

## *Staphylococcus aureus* and antimicrobial resistance: an ongoing threat

**Fig. 2:**
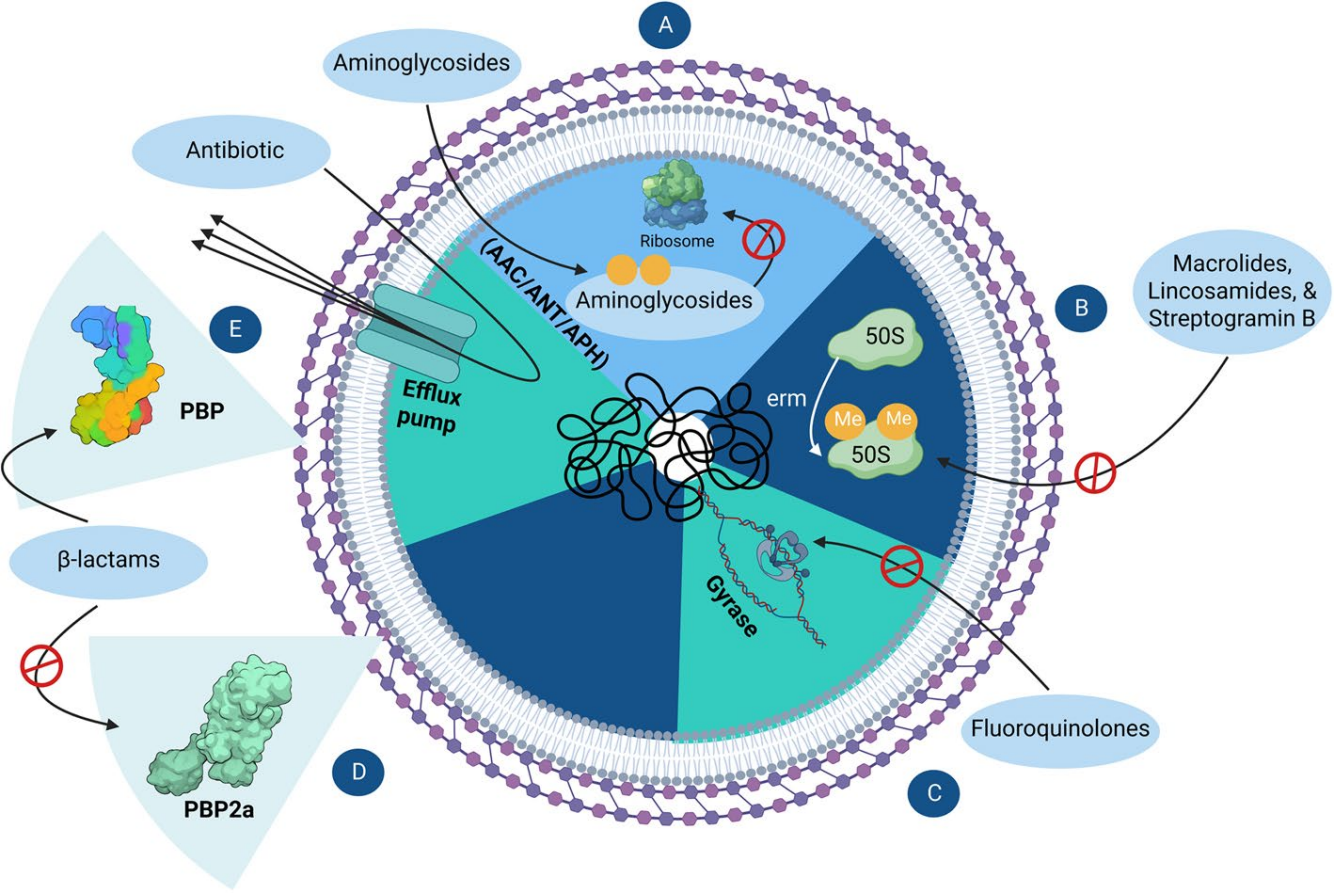
Mechanisms of antibiotic resistance in *S. aureus*. **A** Resistance to aminoglycosides through enzymatic modification by acetyltransferase (AAC), adenylyltransferase (ANT), or phosphotransferase (APH), preventing ribosomal binding. **B** Resistance to macrolides, lincosamides, and streptogramin B through methylation of the 23S rRNA in the 50S ribosomal subunit by erm genes. **C** Resistance to fluoroquinolones due to mutations in the *gyrA* and *gyrB* genes encoding DNA gyrase, thereby reducing drug affinity. **D** Resistance to β-lactams through the *mecA* gene encoding PBP2a, a penicillin-binding protein with low affinity for β-lactam antibiotics. **E** Multidrug resistance mediated by efflux pumps that expel a broad range of antibiotics out of the bacterial cell. These mechanisms collectively contribute to the formidable resistance profile of *S. aureus*, complicating treatment strategies. (Created with https://BioRender.com)

Since the revolutionary discovery of penicillin as the first antibiotic, its extensive overuse and misuse have led bacteria to develop mechanisms that enable them to evade its effects. Remarkably, resistance to penicillin was observed in the 1950 s, merely a decade after it was officially introduced for treating bacterial infections [3]. Since then, *S. aureus* has progressively acquired resistance to successive generations of antibiotics, giving rise to the emergence of the notorious superbug Methicillin-Resistant *Staphylococcus aureus* (MRSA). *S. aureus* demonstrates the ability to confer resistance to a broad spectrum of antibiotic classes through different mechanisms [14, 15], as illustrated in Fig. 2.

One of the fundamental resistance mechanisms contributing to its overall resistance is the presence of multidrug efflux pumps, which are membrane proteins responsible for transporting substances from the cytoplasm to the external environment [16]. Efflux pumps in *S. aureus* can be categorized as either chromosomally encoded, such as NorA, NorB, NorC, MepA, MdeA, SepA, SdrM, and LmrS, or plasmid-encoded, including QacA/B, Smr, QacG, QacH, and QacJ [17–20]. Moreover, *S. aureus* is equipped with molecular mechanisms to resist individual classes of antibiotics. For instance, the acquisition of the *mecA* gene confers resistance to β-lactam antibiotics [21–23]. This gene encodes an altered penicillin-binding protein (PBP), PBP2a, with a markedly low affinity for almost all β-lactam antibiotics. PBP2a differs from the original PBP, which serves as an effective target for binding of β-lactams [21–23].

Additionally, *S. aureus* resistance to fluoroquinolones arises from mutations in gyrase genes (*gyrA* and *gyrB*), which encode essential enzymes involved in DNA replication. Mutations in gyrase genes reduce the affinity of fluoroquinolones for the gyrase protein, rendering them ineffective [24]. Regarding macrolides, lincosamides, and streptogramin B resistance, *S. aureus* utilizes the erythromycin ribosome methylase (*erm*) genes, which encode proteins that methylate adenine residues A2058/2059 in the peptidyl transferase region of domain V of 23S rRNA, thereby altering the binding site and conferring resistance [25]. Additionally, aminoglycosides are inactivated by *S. aureus *via enzymatic modification by acetyltransferase (AAC), adenylyltransferase (ANT), or phosphotransferase (APH), thereby reducing their ability to bind ribosomal targets effectively [26].

### Antivirulence strategies as alternative therapies

The pathogenic process of *S. aureus* involves multiple sequential stages: (a) targeting a suitable host or adhering to appropriate surfaces, (b) colonizing host cells, (c) causing illness and host damage, (d) immune evasion, (e) evacuating from the body, and (f) finding a new host or reservoir [27]. During the exponential growth phase, cell wall adhesive molecules are synthesized to facilitate adhesion and biofilm formation on appropriate surfaces, whereas in the post-exponential phase, toxins and degradative enzymes are synthesized that cause disease and damage host cells [28, 29]. These processes are driven by bacterial virulence factors that represent specialized molecular tools and strategies that enhance bacterial survival, disease, and pathogenicity, without being essential for bacterial viability. Unlike core processes such as cell wall synthesis, protein synthesis, and DNA repair mechanisms [30–32], virulence factors are not required for bacterial survival under non-host conditions. Conventional antibiotics primarily target essential cellular functions, exerting intense selective pressure and accelerating the emergence of AMR, ultimately rendering antibiotics ineffective shortly after their introduction [3].

In contrast, targeting virulence factors aims to disarm the pathogen rather than eliminate it, thereby reducing its ability to cause disease without directly threatening its survival. This approach is hypothesized to impose minimal selective pressure, potentially limiting the development of resistance [24]. By interfering with the expression or function of virulence mechanisms, such strategies offer a promising avenue for attenuating infection severity while preserving the efficacy of current and future antimicrobial interventions.

One of the most essential virulence mechanisms to target in *S. aureus* is its ability to form biofilm. As previously mentioned, biofilm formation can increase bacterial resistance to antimicrobials by up to 1000-fold [9]. This enhanced tolerance arises from multiple synergistic mechanisms. First, the ECM acts as a physical barrier, limiting the penetration of many antimicrobial agents, particularly those with high molecular weight or hydrophobic properties [33]. Second, the unique biofilm microenvironment, characterized by oxygen gradients, low pH, and limited nutrient availability, favors the emergence of metabolically inactive or slow-growing persister cells, which are inherently less susceptible to antibiotics targeting active cellular processes [34]. Third, the high cell density and the presence of extracellular DNA (eDNA) within the ECM facilitate horizontal gene transfer of AMR genes, thereby accelerating the dissemination of resistance determinants within the bacterial community [35]. Additionally, biofilms upregulate specific resistance pathways, including efflux pumps and stress-response regulators, thereby enhancing their survival under antimicrobial pressure [36]. As a result, biofilms have become a focus of antimicrobial research. A deeper understanding of their formation, maintenance, and dispersal mechanisms is crucial for the development of effective anti-biofilm strategies and AMR-counteractive therapies.

### Bacterial biofilms: architects of persistent antimicrobial resistance

Usually mistaken for endospores, which are dormant and non-proliferative bacterial forms [37], biofilms represent a distinct survival strategy in which bacterial cells remain metabolically active while persisting under adverse conditions [38]. The entrapment of bacterial cells within the biofilm matrix confers numerous advantages, including maintaining close spatial proximity that facilitates the exchange of nutrients, signaling molecules, and genetic material [38]. Furthermore, bacterial cells residing in biofilms are shielded from harsh environmental conditions, including temperature fluctuations, nutrient deprivation, and antimicrobial exposure, while retaining the ability to grow and divide [39].

A comprehensive understanding of biofilm architecture and components is essential for developing effective strategies for counteracting their detrimental effects. The ECM of biofilm cells is composed of three main constituents: extracellular DNA (eDNA), extracellular proteins, and exopolysaccharides (EPS) [40, 41]. As previously mentioned, the presence of eDNA within the biofilm ECM promotes horizontal gene transfer among biofilm cells. Additionally, it helps maintain the electrostatic interactions within the ECM, facilitating adhesion and aggregation processes [42]. The release of eDNA occurs due to controlled cell death mechanisms and is regulated by the *cidA* and *lrgA* genes, which are components of the *cidABC* and *lrgAB* operons in *S. aureus*, respectively. These genes play different roles in regulating cell lysis and antibiotic tolerance [43]. Despite the importance of eDNA in biofilm formation and development, *S. aureus* also produces nucleases that regulate eDNA levels in the biofilm environment, thereby balancing biofilm formation and dispersal [44].

Extracellular proteins play pivotal roles in biofilm formation, maintenance, and dispersal. Extracellular proteins facilitate nutrient transport, environmental monitoring via signaling molecules, triggering appropriate cellular responses, and ECM stabilization [45]. EPS plays a central role in maintaining the structural integrity of the biofilm ECM. Due to its complex composition, EPS requires a diverse set of enzymes for complete degradation, thereby keeping the compact structure and mechanical stability of the biofilms. This structural stability helps the biofilm withstand environmental stressors while facilitating nutrient transport to the embedded cells [42, 46].

**Fig. 3:**
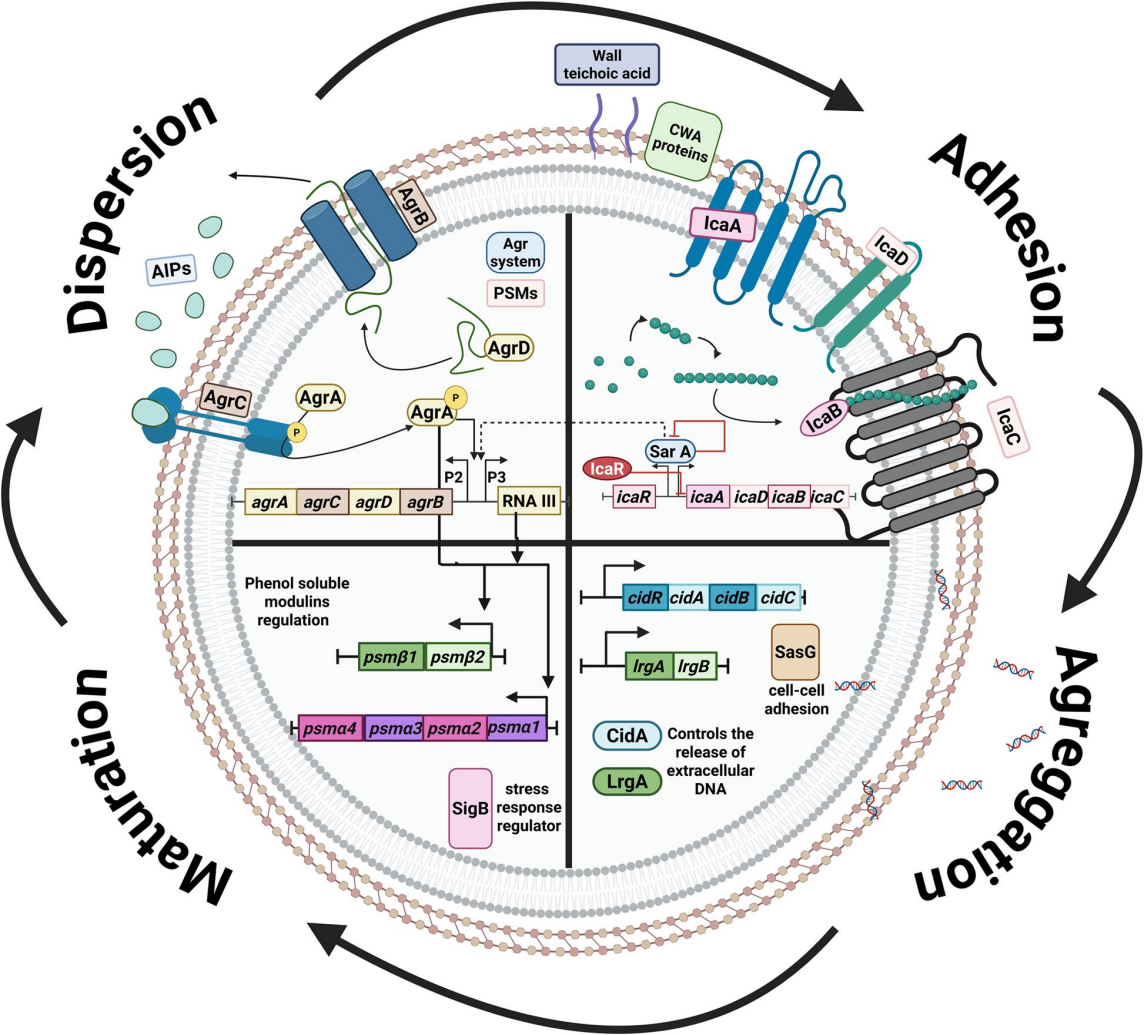
A schematic representation of the biofilm development cycle and its coordination by different regulatory systems in *S. aureus*. The figure demonstrates the four key stages of biofilm formation, illustrated as different compartments of one cell: adhesion, aggregation, maturation, and dispersion. The first stage shows the SarA modulating the adhesion phase along with CWA proteins and teichoic acids. The second stage presents CidA and LrgA regulating the release of eDNA, as well as SasG regulation for cell–cell adhesion and accumulation. The following stage shows the regulation of PSMs in the maturation phase, followed by the final stage of the cycle, characterized by the upregulation of the agr system, modulating biofilm dispersion through proteases, PSMs, and nucleases. The regulation and interconnectedness between the molecular mechanisms are highlighted with interconnected arrows, as SarA self-limits its expression while upregulating the agr system; however, it is labelled with a dashed arrow due to its variable effects discussed in the review. The agr system works in a positive feedback loop and upregulates its own expression and that of the PSMs operons. (Created with BioRender.com)

Biofilm formation proceeds through four main stages: adhesion, aggregation, maturation, and dispersal [40], as illustrated in **(**Fig. 3**)**. Bacterial cells initiate adhesion to biotic or abiotic surfaces via adhesins, which include fimbrial or afimbrial types, such as cell wall-anchored (CWA) proteins [47]. CWA proteins comprise various microbial surface components recognizing adhesive matrix molecules (MSCRAMMs) such as fibronectin-binding proteins (FnBPs), including FnbPA and FnbPB [48], fibrinogen-binding proteins (Fib), clumping factors (ClfA, ClfB) [49], and serine-aspartate repeat family proteins (SdrC, SdrD, and SdrE) [50]. The effects of CWA proteins are primarily regulated by other proteins, such as autolysins, which expose them following peptidoglycan hydrolysis [51]. One of the key components for *S. aureus* attachment also includes wall teichoic acids [52, 53], which are anionic glycopolymer chains attached to the peptidoglycan layer [54].

Following adhesion, the aggregation stage begins. During this stage, cells undergo regulated division in response to environmental conditions [9, 55]. As aggregation progresses, newly formed cells may lose direct contact with the adherent surface; however, they remain interconnected through cell–cell and cell-EPS adhesion, while the bottom layer remains firmly attached, preserving surface adhesion [56]. SasG is a vital surface protein that promotes cell–cell adhesion and accumulation in *S. aureus* via a zinc-dependent adhesion mechanism [57, 58]. Additionally, Polysaccharide intercellular adhesins (PIA), also known as poly-N-acetylglucosamine (PNAG), which are controlled by the icaADBC operon [59], play essential roles in cell–cell and cell-EPS adhesion in staphylococcal biofilms [60]. Another key player is the biofilm-associated protein (Bap), which is essential for adhesion to abiotic surfaces, intercellular adhesion, and biofilm compartmentalization [61, 62]. Despite studies supporting their direct roles in *S. aureus* biofilm formation, a growing number of studies have identified S. aureus biofilm-forming strains that are independent of PIA or Bap proteins, suggesting the presence of complex, regulated systems that affect bacterial adhesion [60, 63, 64].

During the maturation stage, biofilms develop highly organized three-dimensional architectures, with nutrient channels facilitating transport to deeper layers of the cells that lack direct environmental contact [65]. These channels often involve proteins like the Phenol-Soluble Modulins (PSMs), which act as surfactant peptides crucial for shaping biofilm architecture [66]. PSMs are classified into two groups: α-type (PSMα1–4) and β-type (PSMβ1–2), each contributing uniquely to the biofilm physiology [67]. PSMs-α are small, highly amphipathic peptides approximately 20–25 amino acids in length. They possess potent surfactant and cytolytic activities [67], facilitating biofilm structuring by forming channels that promote nutrient flow and mediating biofilm dispersal by disrupting cell-to-cell and cell–matrix interactions during the later stages of biofilm development [10]. In contrast, PSMs-β are longer, approximately 43–45 amino acids, structured as α helices, and exhibit considerably lower cytolytic activity [67]. They contribute to lower biofilm stability by maintaining cohesion between the outer biofilm layers and supporting overall biofilm integrity through interactions with extracellular components [10]. Collectively, these PSM subtypes coordinate to regulate the steady-state balance between biofilm maturation, dispersion, and the persistent colonization of new niches.

The biofilm developmental process involves multiple direct and indirect regulators, such as catabolite control protein A (*CcpA*), which regulates biofilm formation by modulating staphylokinase, an enzyme that converts plasminogen to plasmin to facilitate tissue invasion [68]. Similarly, the ferric uptake regulator (Fur) regulates the expression of extracellular adhesin proteins (Eap) and extracellular matrix binding proteins (Emp), which contribute to biofilm formation in low-iron environments [69]. Additionally, stress-response global regulators such as *SigB* indirectly influence biofilm formation by regulating genes involved in attachment or biofilm maturation in response to environmental stimuli [70, 71].

Finally, the dispersion stage occurs when bacterial cells detect a critical population density within the biofilm ECM. In response, they secrete extracellular proteases, nucleases, and elevated levels of PSMs, thereby disrupting the biofilm matrix [72]. This process results in dispersal of bacterial cells in a more virulent, toxin-producing state, thereby facilitating colonization of new surfaces once high cell densities have been reached. However, if the dispersion stage is disrupted, it leads to persistent biofilms and chronic infections. [73].

Given the protective meshwork of biofilms and their contribution to the AMR crisis, targeting biofilm-forming bacteria would help mitigate the challenges associated with both AMR and biofilm formation on medical devices, hospital surfaces, and beyond [62]. Biofilm disruption can occur at various stages of the biofilm lifecycle, either through inhibiting its formation in early stages or by eradicating preformed biofilms [62]. Treatment strategies include, but are not limited to, natural compounds, nanoparticles, enzymatic treatments, and physical methods. Compounds such as lectin inhibitors are considered early-stage effectors that target carbohydrate-binding proteins to prevent bacterial adhesion [40]. Other compounds focus on interfering with specific stages of biofilm development, such as emodin, an anthraquinone interfering with eDNA production [74], and piperine, which disrupts EPS formation [75], ultimately compromising biofilm integrity and facilitating removal of the biofilm matrix..

Most biofilm-targeting compounds are natural compounds, such as flavonoids, terpenoids, and alkaloids [76], given their diverse mechanisms that act across different stages of biofilm development and target both bacterial growth and the production of virulence factors [77]. Despite the increasing interest in natural compounds as biofilm inhibitors, they are generally not widely recognized as biofilm eradicators, as they require very high concentrations to affect preformed biofilms [78].

This limitation highlights the roles of nanoencapsulation, enzymatic treatments, and physical methods. Nanoencapsulation is a pharmacological approach that enhances the physical properties of drugs by reducing them to the nanoscale, thereby improving their penetration, solubility, and controlled release [79]. It primarily facilitates penetration of the biofilm meshwork and the release of effective compounds. This was previously demonstrated by our research group, where encapsulating thymoquinone, a natural compound derived from *Nigella sativa* with known biofilm-inhibiting activity, enhanced its penetration abilities, thereby enhancing both biofilm inhibition and eradication [80]. Enzymatic treatments disrupt key biofilm components, such as proteases that degrade extracellular proteins and DNases that degrade eDNA, thereby disrupting the biofilm scaffold [9, 10]. The effects of enzymatic treatment can be further enhanced by incorporating enzymes into nanoparticles. Similarly, physical treatments disrupt biofilms by applying external pressure to mechanically degrade the biofilm matrix [38, 39]. Disruption can be achieved using ultrasound, which generates oscillating microbubbles that damage biofilm structure, or laser treatments, which produce focused heat to destabilize the matrix [38, 39].

Despite the in vitro effectiveness of some of these approaches, their application in medical and industrial settings remains limited due to concerns regarding cytotoxicity and target specificity [81]. Accordingly, future research must focus on developing a better understanding of the factors and communication systems that drive biofilm formation, as well as the mechanisms of biofilm dispersion and cell release, to effectively address the AMR crisis and combat bacterial biofilms without triggering resistance patterns similar to those observed with antibiotics.

### Quorum sensing: regulating bacterial communication and pathogenesis

Quorum sensing is the bacterial communication system that enables cells to coordinate the expression of various virulence factors through the production and detection of signaling molecules known as autoinducers, which function analogously to spoken words in human communication [82, 83]. Autoinducers occur in three forms: Acyl-Homoserine Lactones (AHLs), predominantly found in Gram-negative bacteria; Autoinducing Peptides (AIPs), characteristic of Gram-positive bacteria; and Autoinducer-2, a universal signal for interspecies communication [84]. These autoinducers play a crucial role in conveying information about bacterial cell density, indicating whether the bacterial population within the biofilm is sufficiently abundant to initiate dispersal or if it remains too few and should therefore continue dividing before dispersal [85, 86]. However, in cases where biofilm dispersion is unmodulated, persistent biofilm might occur [87].

*S. aureus* possesses approximately 16 distinct two-component systems (TCSs) crucial for sensing population density and regulating downstream responses [88]. These include some extensively studied systems, such as the AgrAC [89], LytSR [90], ArlRS [91], SrrAB [92], SaeRS [93], and AirSR systems [94]. These systems mainly consist of two elements: sensor proteins and response regulator proteins. The sensor proteins are typically membrane-associated histidine kinases that detect environmental changes via signals, such as the autoinducers in quorum sensing [88]. Upon signal detection, these sensor proteins undergo autophosphorylation and subsequently phosphorylate and activate the second component, which are the response regulator proteins. These response regulators subsequently activate or repress specific genes and cellular processes in accordance with the transmitted signal cues [88, 95]. This mechanism enables bacterial cells to adapt to a wide range of environmental challenges, including nutrient limitations, antibiotic stress, membrane integrity disruption, and fluctuations in cell density [96, 97]. By responding to these cues, bacterial populations can make decisions that enhance survival in the host and contribute to pathogenesis. In *S. aureus*, quorum sensing plays diverse roles in the pathogenic cycle [98], regulating the expression of surface proteins involved in adhesion, colonization, and immune evasion, as well as the production of degradative toxins that damage host cells and facilitate nutrient acquisition [98, 99].

### The Accessory Gene Regulator (agr) system

The accessory gene regulator (agr) system in *S. aureus* plays a pivotal role as a highly conserved quorum-sensing mechanism, coordinating the expression of bacterial virulence factors, including toxins, adhesins, and other key elements essential for the pathogenicity of the bacterium in the post-exponential phase [100]. Comprising *agrBDCA* genes, within an auto-regulatory operon, this system actively monitors bacterial cell density and adjusts the expression of both its own components and other target genes accordingly. The system is diverse and is categorized into four agr groups (agr I, agr II, agr III, and agr IV), determined by amino acid polymorphisms in the *agrB*, *agrD*, and *agrC* genes. This categorization results in variations in both agr activity and pathogenic potential among different groups, leading to noticeable differences in hemolytic activity and other virulence gene expression regulated by agr[101]. Moreover, the functionality of the system exhibits significant heterogeneity across different clinical isolates. Loss-of-function mutations within the *agr* operon can impair *agr* activity, consequently affecting the bacterial ability to colonize host tissues [102]. *S. aureus* regulates the agr quorum-sensing system by secreting AIPs tailored to its specific agr type. Four AIP structure classes are found in *S. aureus*, with peptide lengths of 7, 8, or 9 amino acids corresponding to agr types III, I, and IV, and II, respectively.

**Fig. 4:**
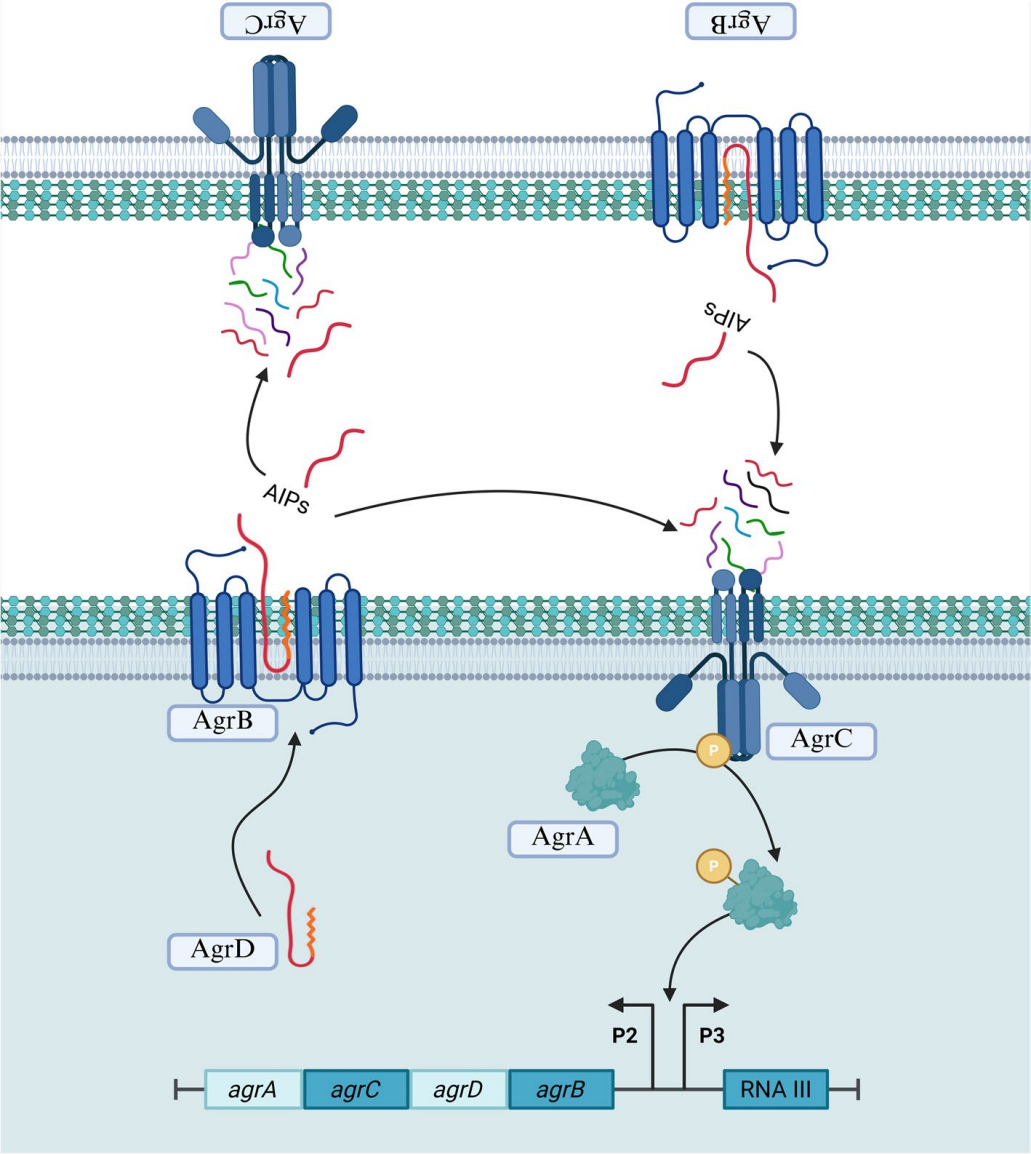
The agr quorum-sensing system initiates a signaling cascade between two bacterial cells, in which autoinducing peptides (AIPs) produced by each cell can cross-activate the histidine kinase receptors of neighboring cells. AgrD, the precursor peptide of AIP, is processed by the AgrB protease, leading to the production of mature AIP. These AIPs accumulate both intracellularly and in the surrounding environment, binding to and activating the histidine kinase receptor AgrC. Upon activation, AgrC undergoes autophosphorylation and then transfers the phosphate group to the response regulator, AgrA. Activated AgrA induces the transcription of RNAII and RNAIII through the P2 and P3 promoters, respectively. Through this regulatory cascade, AgrA upregulates the expression of the agrBDCA operon to enhance further AIP synthesis, while RNAIII upregulates virulence factors and downregulates the expression of cell surface proteins. (Created with BioRender.com)

The agr system in *S. aureus* translates into proteins that orchestrate a complex mechanism, illustrated in **(**Fig. 4), guiding the bacterial transition from a non-invasive, adhesive, commensal state to an invasive pathogenic state [103]. AgrD functions as the precursor for AIPs, which then undergo two cleavage steps. AgrB, acting as a protease, cleaves the C-terminal 18 amino acids of AgrD and facilitates the formation of a thiolactone bond, yielding an intermediate molecule known as AgrD thiolactone [104]. The thiolactone bond intermediate is further processed through a second proteolytic cleavage within the N-terminal leader sequence of AgrD. This proteolysis can be mediated either by SpsB, a type I signal peptidase [105], or by MroQ, another membrane protease regulator, which can substitute for SpsB and facilitate the release of mature AIPs. However, the involvement of MroQ has been reported to occur specifically for agr types I and II. Further experimental investigations are necessary to conclusively identify the responsible protease for this processing step [106]. The two-component system AgrAC subsequently regulates the *agr* operon transcription. The process begins with AgrC, which acts as a receptor that binds to the AIPs. Sensing and activation occur once AIPs concentrations exceed a threshold level, signaling that a high number of bacterial cells are releasing these AIPs. It is as though the bacteria collectively deliver a message to one another, “We, biofilm bacterial cells, have reached a sufficient density that will enable us to spread and colonize the host. Disrupt this meshwork surrounding us so we could disperse and secrete toxins, ultimately inducing damage and disease”. This molecular message triggers structural changes in the histidine kinase domain of AgrC, leading to its autophosphorylation. AgrC then phosphorylates the response regulator AgrA, activating it to promote the transcription of RNA II and RNAIII [107], which are regulated by P2 and P3 promoters, respectively [103]. The P2 promoter precedes the agrBDCA operon, enabling autoregulation of the system, while the P3 promoter enables RNAIII production [108]. In this way, through a precisely coordinated molecular dialogue, *S. aureus* transforms from a harmless commensal into a highly virulent pathogen, ready to breach host defenses and establish infection.

RNA III is a versatile regulatory RNA within the agr system, serving as its primary effector molecule and playing multiple crucial roles in *S. aureus* virulence regulation [109]. RNA III encodes delta hemolysin (hld), a toxin capable of lysing red blood cells. During the early stages of infection, RNA III downregulates the expression of several surface-associated virulence factors while simultaneously activating exoprotein production. This regulation is achieved through both transcriptional and post-transcriptional mechanisms, mediated by the interaction of RNA III with target gene transcripts [110, 111]. Functioning primarily via antisense mechanisms, RNA III binds to target mRNAs to modulate gene expression, either enhancing or inhibiting translation of target genes, depending on the nature of the interactions. Through these diverse actions, RNAIII promotes the translation of genes encoding virulence factors such as alpha-hemolysins (hla), cysteine and serine proteases, and lipases [110], while repressing genes such as staphylococcal protein A (*spa*) gene, which mediates adhesion, coagulase (*coa*) gene, which contributes to host immune evasion, the repressor of toxins (ROT), and LytM, a factor involved in cell wall hydrolase activity [112]. RNA III also directly regulates phenol-soluble modulins (PSMs), which are crucial for biofilm formation and dispersal. PSMs contribute to biofilm architecture by forming nutrient channels and disrupting the biofilm matrix, thereby facilitating bacterial dissemination when required [66]. Additionally, RNA III stabilizes mRNA of MgrA, a global transcriptional regulator that controls biofilm formation by regulating eDNA release, surface protein expression, production of extracellular proteases, and synthesis of PSMs [113]. Furthermore, RNA III controls the expression of the second immunoglobulin-binding protein, thereby enhancing immune evasion and promoting bacterial colonization [114]. This multifaceted regulatory RNA molecule is central to orchestrating the expression of genes associated with virulence, AMR, autolysis, immune evasion, and biofilm formation, ultimately enabling *S. aureus* to respond adaptively to environmental signals, facilitate biofilm dispersal, secrete toxins, and establish successful colonization that drives disease pathogenesis.

### LuxS System

**Fig. 5:**
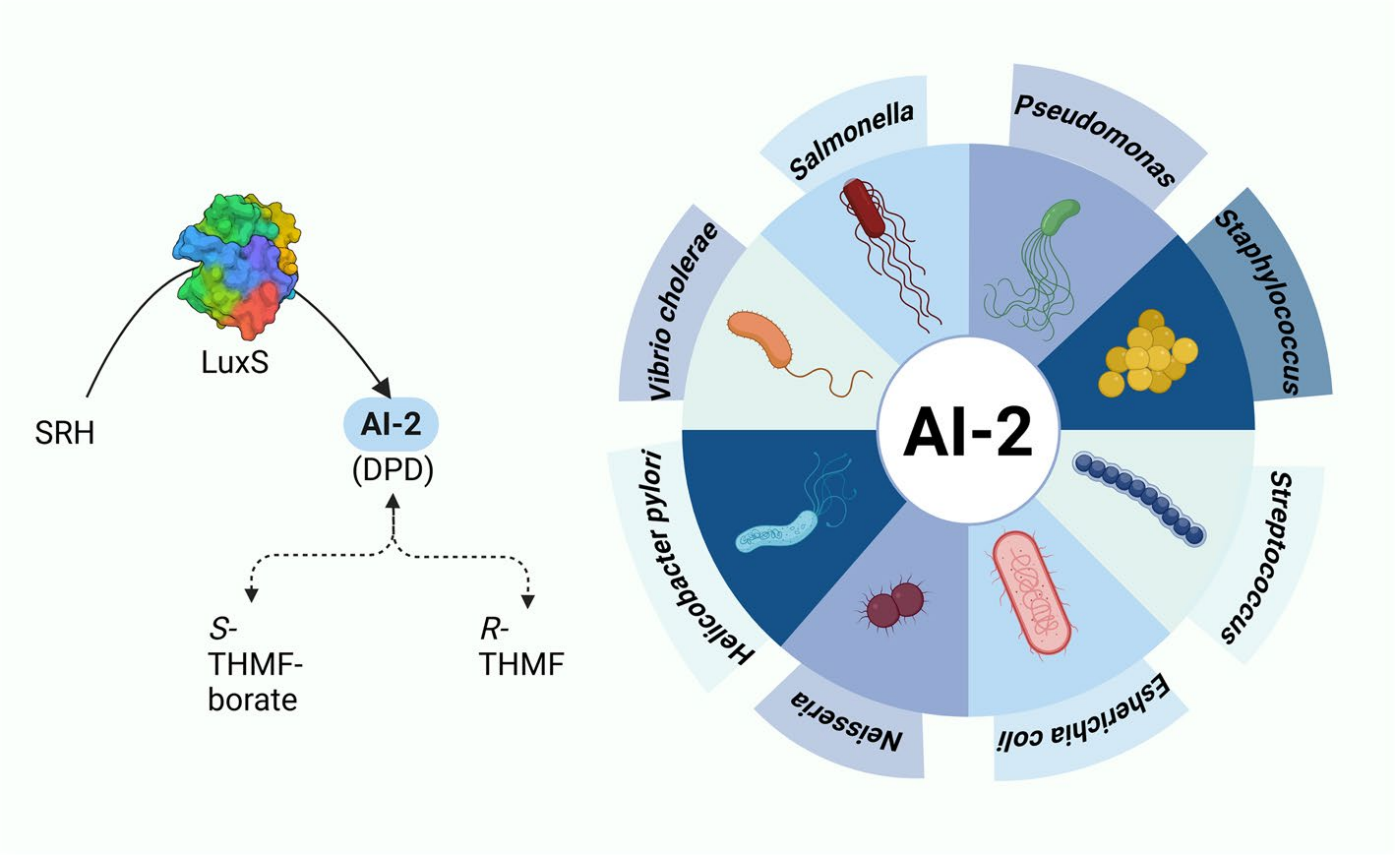
Schematic representation of the universal LuxS communication system and its signaling molecule, AI-2, illustrating interspecies communication among diverse Gram-positive and Gram-negative species. (Created with BioRender.com)

The LuxS quorum-sensing system is widespread among various bacterial species, uniquely shared by both Gram-positive and Gram-negative bacteria, as illustrated in **(**FIG. 5**)**. This system enables interspecies communication, being present in more than 55 species [115]. In *S. aureus,* the LuxS system has been implicated in the active regulation of metabolism, biofilm, and several virulence factors [116, 117]. The system is mediated by autoinducer 2 (AI-2), a signaling molecule produced by the enzyme LuxS as part of the activated methyl cycle (AMC) that represents a fundamental metabolic pathway involved in methyl group transfers, recycling of S-adenosylmethionine (SAM), and the generation of homocysteine [116]. Notably, AI-2 is not a single signaling molecule but a collection of 4,5-dihydroxy-2,3-pentanedione (DPD) derivatives that can convert rapidly to form two epimeric furanoses, (2R,4S)- and (2S,4S)−2,4-dihydroxy-2-methyldihydrofuran-3-one (R- and S-DHMF) [116]. To date, no specific AI-2 receptors have been identified in *S. aureus*. Nevertheless, AI-2 has been shown to directly influence biofilm dynamics by downregulating its formation via activation of the *icaR* pathway. This pathway represses the *ica* operon by facilitating the binding of IcaR to its promoter region [118, 119].

### Is SarA a quorum-sensing system?

The staphylococcal accessory regulator (Sar) family comprises proteins that regulate the expression of various cell wall and extracellular proteins, with roles in virulence and biofilm formation in *S. aureus* [120]. At the forefront of this family is SarA, a DNA-binding protein encoded by the *sarA* locus, which includes three overlapping transcripts, sarA, sarC, and sarB, driven by distinct promoters, P1, P3, and P2, respectively [121].

SarA has long been proposed to influence the agr system, since early work demonstrated that SarA can bind upstream of promoter regions, including those of agr, and modulate expression of virulence determinants in certain strains [122, 123]. However, recent studies reveal that this effect is context-dependent, varying with the strain background, growth phase, and environmental signals. For instance, Zielinska et al. showed that *sarA* mutants from specific clinical lineages maintain near-normal *agr* activity, indicating that *agr* expression can be supported through compensatory regulators [123]. Transcriptomic and proteomic analyses further suggest that SarA primarily represses extracellular proteases and stabilizes surface-associated proteins, and that any effect on agr may be indirect or conditional, rather than universal [124–126].

Beyond its relationship with agr, SarA plays a pivotal role in regulating virulence factors such as α-toxins, PSMs, and Panton-Valentine Leukocidin (PVL) [127], an exotoxin that induces leukocyte lysis [128]. Additionally, SarA represses the production of extracellular proteases, including serine, cysteine, and metalloproteases, as well as staphopain [129]. These proteases are closely associated with severe community-acquired MRSA (CA-MRSA) infections. A recent study has shown that mutations in *sarA* result in a significant increase in overall protease production, a pronounced reduction in biofilm formation, and the accumulation of high-molecular-weight proteins, underscoring the central role of SarA in regulating extracellular protease levels to balance the virulence factor repertoire of *S. aureus* [130].

**Fig. 6:**
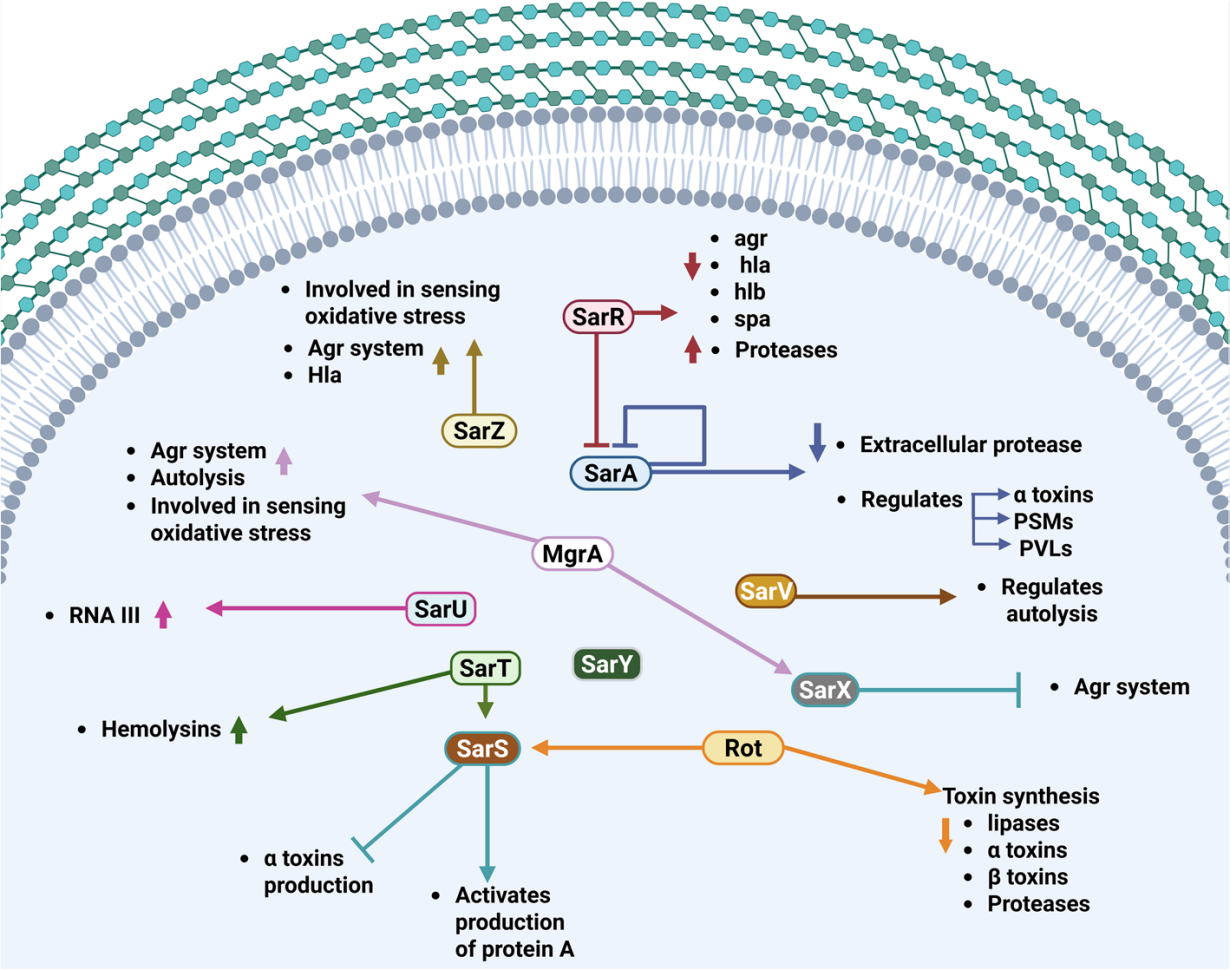
The Sar family proteins exhibit diverse regulatory roles in *S. aureus*, including the modulation of virulence factors, adhesion, and the regulation of other regulatory systems. (Created with https://BioRender.com)

During the exponential growth phase, SarA modulates the post-transcriptional expression of *spa* and collagen adhesion genes by binding directly to their mRNA targets, thereby influencing transcript turnover and stability [131]. The *sar* locus promotes the production of both extracellular proteins, such as hemolysins, and cell wall proteins, including fibronectin-binding proteins [122]. Within the SarA family, homologs are further classified into three subfamilies based on size and structural features: single-domain proteins (*SarA*, -R, -T, -V, -X, and Rot), double-domain proteins (SarS, -U, and -Y), and MarR homologs (MgrA and SarZ) [132] **(**Table 1). These proteins act as transcriptional regulators that either inhibit or enhance each other`s activity, indirectly influencing diverse aspects of bacterial behavior, including virulence, biofilm formation, autolysis, AMR, and metabolic processes [40, 156]. However, they do not directly sense bacterial density or produce AIPs; hence, the Sar system is not considered a quorum-sensing system itself, but rather a global regulatory family of proteins that affect genes related to quorum sensing, such as virulence and biofilm formation genes [157]. The functions of these regulators are summarized in Fig. 6.

**Table 1:** Classification of Sar family proteins and their roles in *S. aureus* virulence regulation

Classification	Protein	Function
Single domain proteins	SarA	● Regulates virulence factors such as α toxins, PSMs, and PVLs [127] ● Negatively modulates the production of extracellular proteases [129] ● Regulates post-transcriptional expression of *spa* and collagen adhesion genes by binding to mRNA targets, affecting turnover and stability [131] ● Activates *agr* operon expression [133]
	SarR	● Suppresses transcription of *sarA* [134], *agr* [133], *hla* [135], *hlb*, and *spa* target genes during post-exponential growth [134] ● Upregulates *aur* (metalloprotease; aureolysin) and *sspA* (V8 protease; serine protease) [136]
	SarT	● Activates *sarS* [137] ● Represses *agr* [138] ● Represses *hla* [138]
	SarV	● Regulates autolysis [139]
	SarX	● Controlled and activated by MgrA and acts to repress *agr* and its target genes [140, 141] ● Upregulates *ica* operon expression and PIA production [142] ● Negatively regulates the *agr* system [141]
	Rot	● Upregulates *sarS* [143] ● Negatively regulates toxin synthesis, including lipases, α toxins, enterotoxins, and various forms of proteases [144, 145]
Double domain proteins	SarS	● Activated by Rot to repress alpha toxin production [146] ● Activates protein A synthesis [146]
	SarU	● Positively regulates the agr system by indirectly upregulating RNAIII expression [147]
MarR homologs	MgrA	● Regulates alpha-toxins, proteases, and capsule production [148, 149] ● Regulates the expression of *sarZ* [150] ● Regulates NorA and NorB efflux pumps [151] ● Repress surface proteins as SasG [152] ● Controls and activates SarX function [141] ● Involved in sensing oxidative stress [153]
	SarZ	● Represses SarA and activates MgrA [154] ● Activates the agr system [155] ● Regulates hemolysins (*hla* and *hlb*), *spa,* and proteases production [155] ● Involved in sensing oxidative stress [153]

### Significance of Quorum Sensing in Virulence, Biofilm Formation, and Antimicrobial Resistance

**Fig. 7:**
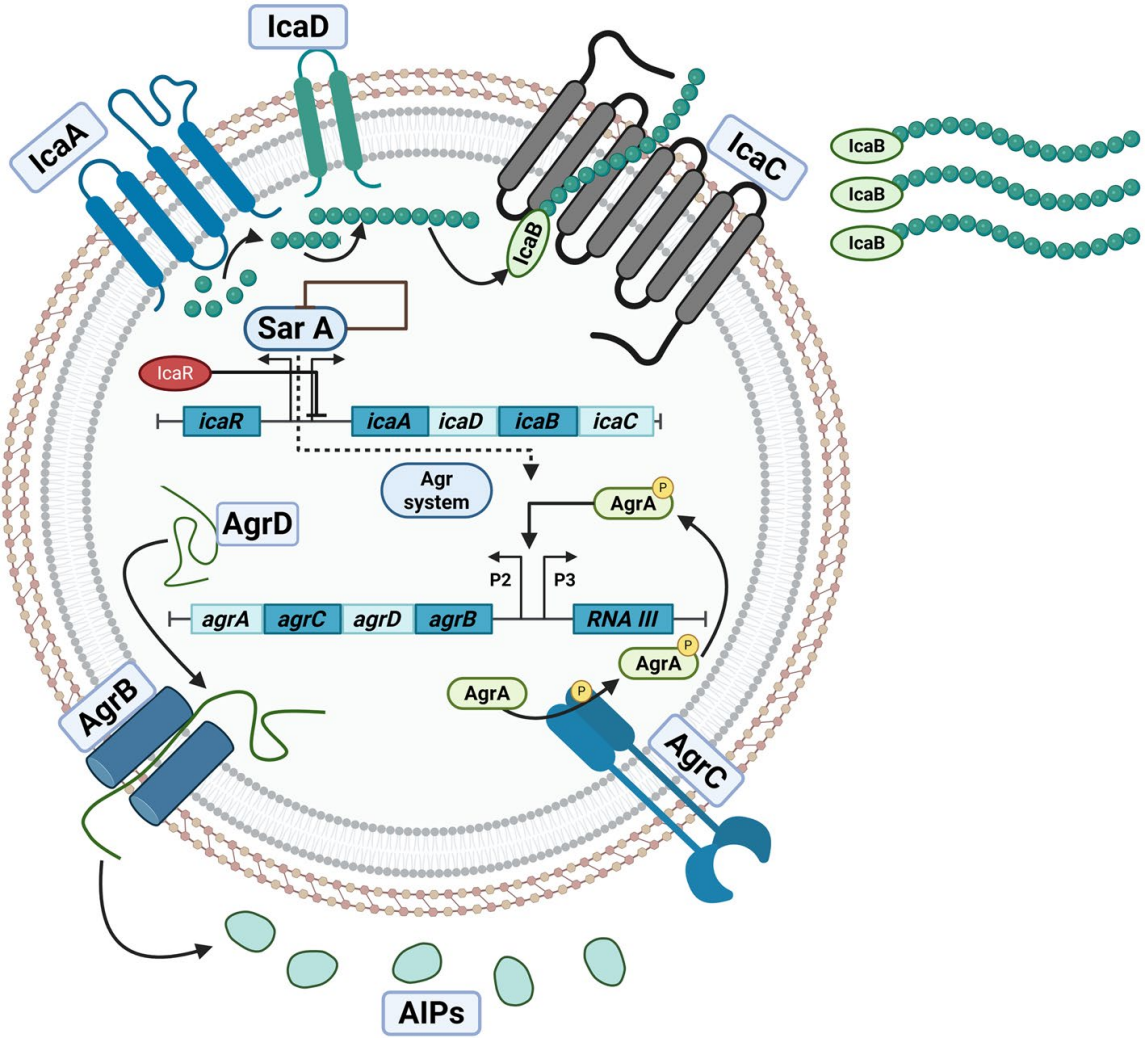
Quorum sensing serves as a central hub connecting different regulatory systems in *S. aureus*. At low cell density, SarA upregulates the icaADBC operon to promote bacterial adhesion. In contrast, at high cell density, SarA downregulates its own expression and, in some studies, has been reported to activate the agr system, which, when activated, promotes biofilm dispersal and virulence factor production. (Created with BioRender.com)

Quorum sensing plays a crucial role in regulating virulence factors production, biofilm formation, and AMR, all in response to bacterial cell density [158]. At low cell densities, characterized by low concentrations of autoinducers, global regulators promote the expression of surface proteins that facilitate adhesion and establish initial colonization sites [159]. Concurrently, toxin and extracellular protein production are suppressed to prevent early detection by host defenses when bacterial numbers are insufficient for effective confrontation [82]. In the context of biofilm formation, quorum sensing activates the MSCRAMMs, cell wall-anchored (CWA) proteins, and genes encoding PIAs, autolysins, and enolases. These elements collectively contribute to the formation of the extracellular matrix that embeds and protects bacterial communities [160]. The biofilm cycle is a complex, multi-stage process regulated by various interconnected regulatory systems, as illustrated in (Fig. 7**)**, which depicts the interplay between the *sarA*, agrBDCA, and icaADBC operons. At low cell density, SarA upregulates the icaADBC operon, promoting biofilm formation through enhanced bacterial adhesion [110]. On the other hand, at high cell density, sensed by elevated AIP levels, SarA exerts a self-limiting effect and, in conjunction with the activated agr system, facilitates biofilm dispersal and triggers expression of secreted virulence factors, leading to tissue damage and disease progression [161]. Additionally, genes responsible for forming channels within biofilms and genes encoding dispersal factors are upregulated, promoting bacterial spread within the host [162].

Quorum sensing is essential not only for biofilm formation and toxin regulation but also for regulating transporters [163] and efflux pumps [164]. Consequently, quorum sensing contributes directly and indirectly to AMR. It helps indirectly by its role in biofilm formation, which acts as (1) a barrier against antibiotic penetration [165], (2) a hiding scaffold from host immune cells [166], (3) a medium for AMR genes transfer [167], and (4) antimicrobial compounds degradation [7, 165]. As well, it helps directly through the strong correlation between quorum sensing and induced expression of AMR genes [164], as evidenced by the overexpression of RNA III and *sarA* genes in highly resistant *S. aureus* strains compared to sensitive ones [168]. However, it remains unclear whether bacterial cells first developed resistance that subsequently stimulated quorum sensing, or whether quorum sensing initially triggered the development of resistance. It is also worth noting that other studies have correlated agr dysfunction with increased resistance to the gold-standard antibiotic vancomycin [169–172]. Moreover, Fowler et al. proposed that *agr* dysfunction leads to persistent bacteremia resulting from intermediate-level glycopeptide resistance (GISA) [169], which is a class of antibiotics including vancomycin, teicoplanin, and ramoplanin [173]; however, staphylococcal persistence resulting from *agr* dysfunction can be attributed to increased expression of adhesion and reduced expression of extracellular proteases, too [169]. Differences in agr groups can also affect virulence and biofilm formation; Derakhshan et al. [174] found that isolates from the agr III group were more virulent than those from the agr I group; moreover, they found that resistant strains were more commonly found in agr-deficient strains than in active ones. Accordingly, this area of study requires further investigation to elucidate the relationship between quorum sensing and AMR mechanisms.

### Mechanisms of action of quorum sensing inhibitors in *S. aureus*

Given the crucial role of quorum sensing in the pathogenic life cycle, quorum-sensing inhibitors (QSIs) have emerged as promising therapeutic strategies for combating severe bacterial infections characterized by elevated toxin production and virulence. Studies on *S. aureus* mutants lacking key quorum-sensing genes have demonstrated reduced virulence in mouse models of skin infections and pneumonia [175–177]. However, the efficacy of QSIs may be limited in chronic infections, where biofilm plays a central role. This limitation stems from the fact that the ultimate goal of quorum sensing is to initiate biofilm dispersal upon reaching a critical cell density; therefore, disrupting this process may promote biofilm persistence [12].

**Fig. 8:**
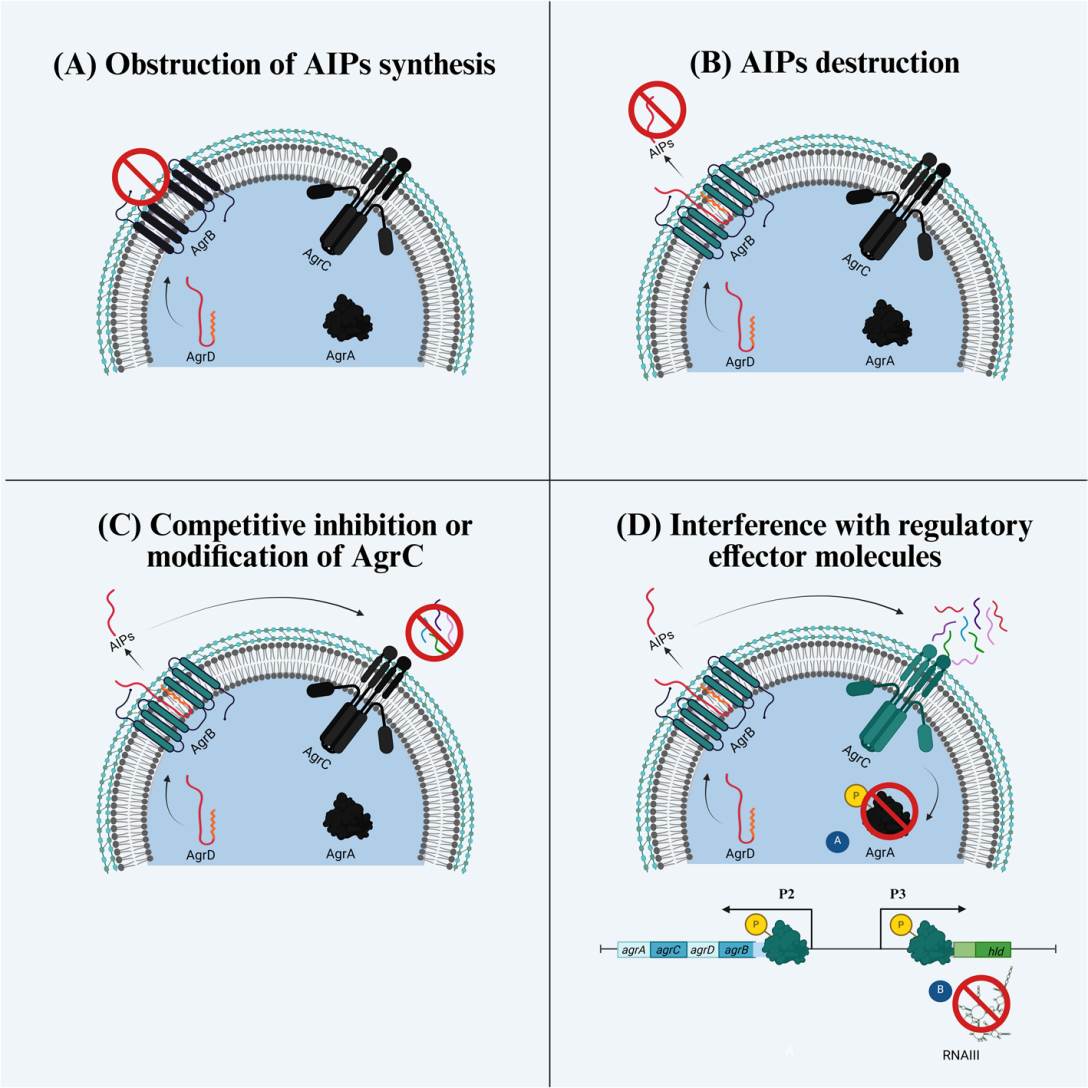
Quorum sensing can be inhibited at multiple stages throughout its signaling cycle. Potential targets include (**A**) blocking AIP synthesis, (**B**) degrading synthesized AIP molecules, (**C**) competitively inhibiting AgrC or modifying its receptor-binding site, and (**D**) interfering with the main downstream regulatory molecules. System components highlighted in black indicate the specific points at which inhibition occurs, thereby stopping signal progression. (Created with BioRender.com)

A comprehensive understanding of quorum sensing in biofilm development is essential for designing targeted therapeutic strategies. In this context, a clear distinction must be made between QSIs and anti-biofilm agents, as they constitute discrete molecular classes with fundamentally different mechanisms of action. As illustrated in Fig. 8, QSIs act on various components of the quorum-sensing system, any of which, when disrupted, can disrupt the entire signaling cascade [178]. Their mechanisms include: (A) inhibiting AIP synthesis, (B) degrading existing AIPs, (C) competitively inhibiting and modifying quorum-sensing receptors, and (D) interfering with downstream regulatory pathways [179, 180]. Quorum sensing can also be inhibited indirectly by regulators such as SigB and two-component system (TCS) proteins [86]. In contrast, anti-biofilm agents primarily target biofilm-specific processes, such as blocking adhesion molecules essential for biofilm initiation or enzymatically degrading biofilm matrix components, including exopolysaccharides, proteins, and eDNA [181–183]. Although both classes of therapeutics modulate biofilm behavior, they act through distinct molecular targets and therapeutic mechanisms. Antibiofilm agents aim to inhibit or eradicate existing biofilms, whereas QSIs primarily lead to persistent biofilms. The following section details the diverse mechanisms of QSIs, with representative studies, summarized in Table 2.

**Table 2:** Examples of QSIs, Their Sources, and Mechanisms of Action

Compounds	Source category	Source description	Mechanism of action	Reference
*Staphylococcus simulans* AIPs	Bacteria	*S. Simulans*	Competitive inhibition of AgrC	[184]
Pyocyanin		*Pseudomonas aeruginosa*	Interference with regulatory molecules (AgrA)	[185]
Acylases (PvdQ)		*Pseudomonas aeruginosa*	Destruction of AIPs	[186]
ω-Hydroxyemodin	Fungi	*Pestalotiopsis restrictum*	Interference with regulatory molecules (AgrA)	[187]
Ambuic acid		*Pestalotiopsis neglecta*	Obstruction of AIPs synthesis	[188]
Lacases		*Pleurotus ostreatus*	Destruction of AIPs	[178]
Hispidulin	Plants	Tamarix Bark	Interference with regulatory molecules (AgrA)	[189]
Baicalin		*Scutellaria baicalensis*	Interference with regulatory molecules (AgrA)	[190]
Ajoene		Garlic	Interference with regulatory molecules (RNAIII)	[191]
Polyhydroxyanthraquinones		*Rubia tinctorum*	Obstruction of AIPs synthesis	[192]
Oleanene		*Ligustrum lucidum*	Obstruction of AIPs synthesis	[193]
Solonamide A and B		*Photobacterium halotolerans*	Obstruction of AIPs synthesis	[194]
Staquorsin	Synthetic	Synthetic	Interference with regulatory molecules (AgrA)	[195]
Hydroquinone		Synthetic	Interference with regulatory molecules (RNAIII)	[196]
Savirin		Synthetic small molecule	Interference with regulatory molecules (AgrA)	[197]

### Obstruction of autoinducers synthesis

Obstruction of AIPs synthesis can occur at multiple stages and target different molecules; mainly AgrB and AgrD. Modifications in the C-terminal tail of AgrD can prevent its normal cleavage, thereby disrupting the crucial interaction between AgrB and AgrD [198]. Targeting AgrB is another potential strategy to block the AIPs synthesis. Studies introducing random mutations into AgrB have shown that many of these mutations abolished its peptidase activity, thereby preventing AgrD processing and effectively stopping the AIP production cycle [199]. Such disruptions, whether impairing AgrB function or its interaction with AgrD, ultimately prevent the generation and extracellular accumulation of AIPs. This, in turn, inhibits activation of the sensor kinase AgrC and suppresses the downstream virulence cascade [200].

However, targeting AIPs synthesis in *S. aureus* poses significant challenges due to the structural variability of autoinducers and the existence of 4 distinct agr types. Effective targeting should be highly specific to the AIP structure and its precursor AgrD, restricting potential interventions to particular agr types and necessitating prior agr typing. Notably, only one step in AIP synthesis is conserved across all agr types: the cleavage of AgrD by AgrB [200]. Despite sequence variations among AgrB proteins from different agr groups, the first 34 N-terminal residues located in the cytoplasm, along with His-77 and Cys-84 residues crucial for the proteolytic activity, are highly conserved [201]. Targeting these conserved regions offers the possibility of broadly inhibiting all agr types [202]. One study demonstrated that aprotinin, a serine protease inhibitor, reduced RNAIII levels in *S. aureus* by inhibiting the protease activity required for AIP processing and maturation [203]. Another approach involved vaccination with hapten-linked AIP-4, which exhibited high antigenicity, enabling the production of monoclonal antibodies against AIP-4. This conferred passive immunity and led to attenuation of virulence. However, the antibodies generated were specific to agr IV strains [204], limiting their applicability as a broadly effective therapy.

### Autoinducers destruction

Disrupting quorum sensing by degrading autoinducers after their production is known as quorum quenching [12]. This approach offers an alternative anti-virulence strategy that bypasses the structural specificity challenges associated with targeting AIPs synthesis or receptor binding. Rather than blocking AIP production or competing for receptor sites, quorum quenching degrades or inactivates AIPs directly, thereby silencing the quorum-sensing cascade by eliminating the signaling molecules before they can bind to their cognate receptor, AgrC [205, 206].

Enzymatic degradation is among the most extensively studied routes to the destruction of autoinducers. While initially studied in Gram-negative bacteria, this strategy is increasingly recognized for its potential in Gram-positive pathogens such as *S. aureus*. Enzymes, including lactonases, acylases, oxidoreductases, and proteases, have been shown to disrupt quorum sensing by cleaving or modifying key functional groups in the AIP structure [207]. For instance, the AiiA lactonase, identified in *Bacillus thuringiensis*, hydrolyses the thiolactone ring of AIPs, a structural feature required for AgrC binding, and has been shown to significantly reduce hemolysin production and biofilm progression in *S. aureus* by degrading AIP-I [208, 209]. Similarly, AiiM, an enzyme from *Bacillus cereus,* has demonstrated potent quorum-quenching effects in vivo, reducing tissue damage and lesion formation in a murine skin infection model [178]. Additionally, Esp, a protease secreted by *Staphylococcus epidermidis (S. epidermidis)*, has been shown to degrade AIPs into non-functional fragments, thereby inhibiting *agr* signaling and reducing virulence in *S. aureus* [210].

In addition to exogenous enzymes, endogenous proteases secreted by *S. aureus* modulate agr activity by affecting AIP availability and stability, rather than by directly cleaving mature AIPs. One example is aureolysin, a zinc-dependent metalloprotease that has been shown to indirectly affect AIP-mediated signaling by degrading components such as AgrD and other extracellular intermediates involved in AIP maturation [211, 212]. This leads to reduced RNAIII expression and virulence attenuation.

Commensal species also contribute to quorum quenching by producing proteases or metabolites that degrade or destabilize AIPs. For example, *S. epidermidis* has been shown to limit *S. aureus* colonization and virulence not only through competitive inhibition, but also by secreting enzymes and matrix components that reduce the stability or effectiveness of AIPs in mixed microbial environments [203, 210, 213]. Host immune environments, particularly inflamed or infected tissues, are also rich in proteolytic enzymes like neutrophil elastase and matrix metalloproteinases (MMPs), which nonspecifically degrade extracellular peptides. Although direct evidence of host-derived AIP degradation remains limited, these proteases may act synergistically with therapeutic interventions, synthetic biology, and enzyme engineering to disrupt quorum sensing [214, 215]. While quorum-quenching enzymes are not yet widely available for clinical use, directed evolution and structure-based design have shown promise in expanding the substrate specificity of existing quorum-quenching enzymes, which were previously active against AHLs, to include AIPs [209, 216].

### Competitive inhibition and modification of quorum-sensing receptors

Competitive inhibition is a phenomenon that occurs when a non-specific molecule occupies the active site of an enzyme, thereby preventing the substrate from binding and disrupting biological processes that depend on substrate–receptor interactions. In the context of *S. aureus*, competitive inhibitors targeting the agr quorum-sensing system have been shown to reduce abscess formation in soft-tissue infections [204, 217, 218], highlighting the potential of quorum-sensing inhibitors to mitigate infection severity.

Interestingly, *S. aureus* itself can interfere with the quorum sensing of other *S. aureus* strains through a phenomenon known as agr interference. In this process, AIPs produced by one agr subtype competitively inhibit the agr system in *S. aureus* strains belonging to different subtypes [219]. Agr interference is characterized by cross-inhibition among several groups, notably between AIP-I and AIP-IV, due to their structural similarity [220], as well as among AIP-II and AIP-III [101, 221]. For instance, AIP-I typically activates its corresponding receptor, AgrC-I, in agr subtype I strains but competitively inhibits the non-corresponding receptors, AgrC-II and AgrC-III, in agr subtypes II and III, respectively [221].

Current research efforts aim to develop universal competitive inhibitors effective against *S. aureus* agr subtypes. This can be achieved by structurally modifying AIPs to create competitive analogs, either by incorporating different moieties or altering the thiolactone scaffolds [204, 217, 218, 222]. Truncated AIPs analogs, such as trAIP-1, tr-AIP-2, and tr-AIP-4, have demonstrated potent inhibitory effects across strains of different agr subtypes [204]. The approach of designing synthetic analogs based on original AIPs is notable for its biological relevance and molecular stability [223]. For instance, Lyon et al. evaluated the stability of several macrocyclic peptide analogs and confirmed their resistance to degradation by multiple endoproteases and their efficacy in reducing dermonecrotic lesions in vivo infections caused by diverse *S. aureus* strains [221]. Despite their potent activity, these macrocyclic peptide analogs revealed no significant impact on bacterial viability, and repeated exposure did not result in the emergence of resistance [221]. These findings support the advancement of such analogs into preclinical evaluation and highlight the potential of alternative strategies aimed at virulence attenuation.

There is also a growing interest in macrocyclic peptides derived from other bacterial species as potential quorum-sensing inhibitors. For instance, solonamide A and solonamide B, isolated from a marine photobacterium, have demonstrated inhibitory activity against the agr system [224]. Structural analysis suggested that these solonamides resemble truncated AIPs, such as tr-AIP-2 and tr-AIP-3, and may function as competitive inhibitors of the *S. aureus* AgrC receptor [224]. Although specific IC50 values were not reported, additionally, northern blot analysis confirmed the agr-interfering activity of the solonamides in both *S. aureus* strain 8325–4 and the highly virulent CA-MRSA strain USA300 [224].

To date, limited research has focused on developing different therapeutics that mechanistically alter the structure and conformation of quorum-sensing receptors, particularly AgrC. Nevertheless, several studies have highlighted the significant impact of structural modifications of AgrC on quorum sensing and virulence regulation [225]. For instance, Jensen et al. introduced targeted mutations into the first transmembrane segment and extracellular loops of AgrC in *S. aureus*. By substituting three specific amino acid residues in Loop 2 of AgrC1 and AgrC4, they reversed AIP specificity, transforming AIP‑1 from an antagonist to an agonist and vice versa [226]. While mutations in Loop 1 did not affect ligand recognition, they were essential for full receptor activation. These findings highlight that minor changes to membrane topology or ligand-binding loops, such as Loop 1 or Loop 2, can disrupt AIP recognition or impair signal transduction [226].

Moreover, variations in the extracellular sensor domain of AgrC are highly responsible for the diversity in AIP recognition among agr types. Studies constructing receptor chimaeras by swapping AgrC domains between agr groups have demonstrated alterations in specificity and signal response, underscoring the modularity and adaptability of AgrC to targeted modifications [227]. Beyond the extracellular domain, modifications in the intracellular kinase domain of AgrC can also impair QS. For instance, a single amino acid substitution of tyrosine to cysteine at position 223 of AgrC disrupts the interaction between the AgrC histidine kinase and AgrA, blocking its phosphorylation and downstream signaling [228]. A similar substitution can interfere with the quorum-sensing cascade, thereby reducing virulence gene expression and increasing the production of bacterial surface proteins. Additionally, the naturally occurring K73I mutation in the cytoplasmic loop of AgrC, identified in clinical isolates, has been linked to delayed *agr* activation and reduced expression of virulence genes, such as α-hemolysin [229]. Other point mutations, including R238H and H239Q in the DHp (dimerization and histidine phosphotransfer) domain, have been shown to disrupt AgrC autophosphorylation, thereby preventing signal transduction to AgrA and downstream RNAIII expression [230].

### Interference with regulatory molecules

Understanding how AgrA and RNA III regulate *S. aureus* pathogenesis provides critical insights for designing strategies to target these regulators and thereby directly suppress virulence gene expression. Targeting AgrA often focuses on its DNA-binding domain, which is responsible for transcriptional activation at the p2 and p3 promoters. This domain corresponds to the C-terminal region of the conserved LytTR domain, known as AgrAC [231, 232]. Several AgrA inhibitors have been developed to bind specific regions within this domain, including Tyr229 [232] and various hydrophobic residues in the AgrA active site [197].

For instance, studies have demonstrated that diflunisal binds to the C-terminal domain of AgrA [233], whereas molecules such as savirin [197] and bumetanide [232] specifically interact with the Tyr229 residue in AgrA. Inhibiting AgrA disrupts quorum sensing and leads to the downregulation of key virulence factors, including PSMs, hemolysins, and RNA III. Notably, Palaniappan et. al reported that AgrA inhibitory compounds reduced ulcer progression in a mouse dermonecrosis model, with bumetanide exhibiting additional anti-inflammatory effects [232]. Beyond savirin and bumetanide, other small molecules such as staquorsin, a triazoloquinazoline analog, have shown similar mechanisms by inhibiting the AgrA–DNA interaction and reducing Agr-controlled virulence expression [195].

Natural compounds have also emerged as potential AgrA inhibitors. For instance, physalin derivatives, particularly physalin H, B, and I, have been identified to bind near key residues of the AgrA LytTR domain, including L186, N201, and R198. These interactions result in decreased transcription of α-hemolysin and biofilm-associated genes in MRSA [234]. Another strategy for targeting AgrA is the use of antisense oligonucleotides directed against agrA transcripts, thereby reducing its translation [200]. Studies employing locked nucleic acid (LNA) antisense constructs have demonstrated significant reductions in AgrA levels and a corresponding decrease in toxin production in both MRSA and methicillin-sensitive clinical isolates. Similarly, small interfering RNAs (siRNAs) targeting *agrA* and *sarA* have caused markedly inhibited hemolytic activity and biofilm formation [235].

An alternative approach to disrupting quorum sensing involves targeting RNAIII, the primary effector molecule of the agr quorum-sensing system. Regulatory RNAs, such as RNAIII, orchestrate post-transcriptional expression of numerous virulence factors, including hemolysins, proteases, and exotoxins [236, 237]. Unlike some inaccessible protein targets, RNAIII can be targeted through chemical methods such as antisense oligonucleotides, siRNAs, or RNAIII-inhibiting peptides (RIPs), which interfere with its regulatory and structural functions [238, 239]. However, several challenges limit the therapeutic use of RIPs, including peptide stability and activity, which hinder their clinical potential. Advances in delivery strategies may enhance RIP stability and bioavailability, enhancing their effectiveness [109]. Additionally, previous findings indicate that functionally designated antisense RNAs that complement specific sequences in RNA III can reduce its activity, thereby lowering α-hemolysin production and host-cell cytotoxicity [240]. Additionally, synthetic RNA decoys that mimic RNAIII-binding partners have been proposed as a means to sequester RNAIII, preventing it from interacting with its mRNA targets and thereby attenuating virulence gene expression [241].

### Challenges and limitations of quorum-sensing inhibitors

Despite the promising potential of QSIs, several limitations arise. One of which is the biofilm persistence after QSIs intervention. The agr system in *S. aureus* usually functions in the later phases of the biofilm cycle to allow biofilm dispersion; accordingly, disrupting this cycle strongly affects the dispersion process, leading to the persistence of biofilms that require further intervention for eradication [83]. Additionally, outliers to normally functioning agr in *S. aureus* would appear. Namely, agr-defective or agr-mutant strains are typically found in chronic infections, as *S. aureus* often undergoes mutations during disease progression [200]. With such strains, QSIs alone would be inefficient, suggesting that the treatment model could allow *S. aureus* to gain an advantage in the progression from the acute to the chronic stage.

Furthermore, differences in agr systems notably complicate the use of QSIs due to variations in canonical AIPs, requiring pre-testing for infection specificity. Beyond this, the timing of treatment administration (early-stage or late-stage) is an essential factor that should be considered and further complicates delivery [200]. Finally, the ability of *S. aureus* to develop varying resistance mechanisms against antibiotics serves as an alert that warrants careful consideration, as the bacterium could potentially adapt to QSIs. This could be achieved by altering virulence production, employing agr-independent gene expression, or acquiring alternative resistance mechanisms [242]. These limitations complicate the application of QSIs due to the necessity of monitoring post-treatment physiological, phenotypic, and adaptive bacterial responses; tackling variation within agr systems; addressing the complexities of targeting through various delivery systems; and evaluating the pharmacokinetics, pharmacodynamics, and resistance patterns of QSIs [243].

### Biofilm persistence following agr inhibition

While QSIs show promise in attenuating virulence in *S. aureus*, their use presents a critical unintended consequence. Several studies have demonstrated a correlation between the agr activity and biofilm dispersion, showing that agr-deficient *S. aureus* strains form stronger biofilms and exhibit enhanced persistence compared to wild-type strains [83]. Under normal conditions, the agr system modulates a regulatory shift during biofilm dispersion by upregulating proteases and downregulating adhesins [244]. Inhibition of agr disrupts this transition: protease expression remains low, and adhesion proteins continue to be expressed, leading to sustained biofilm attachment and impaired dispersion [245].

Consequently, although agr inhibition effectively reduces virulence factor expression, it unexpectedly promotes biofilm persistence, enhancing the ability of *S. aureus* to persist and potentially contributing to chronic infections [246]. Park et al. found that dysfunctions within the agr system were associated with persistent *S. aureus* bacteremia [247], while Schweizer et al. found that infection with agr-inhibited strains in critically ill patients was associated with a 91% increase in mortality rates, primarily due to persistent infections and increased resistance to antibiotics such as vancomycin [170].

### QSIs ineffectiveness against agr-independent and agr-defective mutants

The broad application of QSIs across different *S. aureus* strains may be limited by substantial genetic diversity [248]. For instance, QSIs are ineffective against agr-deficient strains, which inherently lack a functional agr system. Although these strains may exhibit a diminished ability to initiate new infections, agr dysfunction often arises through spontaneous mutations or metabolic suppression during disease progression [249]. Interestingly, agr-deficient strains tend to form persistent biofilms and are resistant to QSIs [250]. Moreover, such mutations are predominantly found within biofilm-associated populations of S*. aureus* [243]. The absence of functional agr signaling in these communities confers a survival advantage by promoting immune evasion and facilitating the establishment of dense, persistent biofilms [243]. As a result, the effectiveness of QSIs is mainly limited to planktonic bacterial populations, where they can downregulate virulence factors involved in colonization and disease progression without inducing persistent biofilms. However, this narrow scope significantly restricts their clinical utility, particularly given that biofilms are implicated in approximately 80% of chronic infections, according to the National Institute of Health (NIH) [251].

Furthermore, the mechanisms underlying biofilm formation in *S. aureus* exhibit considerable variability. Certain strains rely on biofilm-associated proteins such as Bap, which facilitates early bacterial adhesion [252]. The expression of bap is regulated by SarA via an agr-independent pathway, highlighting that biofilm development in these strains may not exclusively rely on quorum sensing [253]. Given these complexities, further research is needed to develop alternative or complementary therapeutic strategies that address the limitations posed by agr-deficient strains and those utilizing agr-independent pathways for biofilm development.

### Complexities in targeting quorum-sensing pathways in *S. aureus*

Targeting activation sites with QSIs is a complex process with limitations that require selectivity and specificity for the intended molecular targets [254]. The challenge is further complicated by the presence of four distinct agr types, each defined by its own canonical AIP [255]. The use of QSIs across diverse *agr* types often results in inhibition of one or two types, as some agr variants may not respond to a general AIP antagonist. Thus, developing inhibitors capable of effectively blocking all four types require further extensive research investigation [200]. Lacking the selectivity and specificity in QSIs also risks off-target effects on commensal bacterial populations [256]. Many commensal bacteria in the human microbiome rely on quorum-sensing mechanisms, including AIPs that regulate interspecies communication and maintain microbial homeostasis [257]. Thus, disruption of these signaling pathways by non-specific QSIs could lead to dysbiosis, impairing beneficial bacterial communities and potentially facilitating disease through the overgrowth of opportunistic pathogens [258].

The primary aim of using QSIs is to attenuate the virulence of *S. aureus* by disrupting the agr system signaling. However, several virulence factors in *S. aureus* are regulated independently of the agr system. These alternative regulatory pathways include systems such as SrrAB and SaeRS [242]. Furthermore, the host environment and specific sites of infection introduce additional variability, such as oxygen levels, pH, and different immune responses, which can influence both *agr*-dependent and *agr*-independent gene expression [259]. Taken together, these insights highlight that virulence regulation in *S. aureus* is controlled by multiple overlapping pathways, indicating that targeting the agr system alone, without accounting for these alternative regulatory mechanisms, may be insufficient for comprehensive virulence attenuation.

### Resistance mechanisms against quorum-sensing inhibitors

Although not widely reported, some bacterial populations have evolved strategies to mitigate the effects of quorum-sensing inhibitors (QSIs). These mechanisms include activation of efflux pumps, biofilm-driven resistance, increased production and structural modifications of AIPs, and mutations in key quorum-sensing components, including receptor proteins [260]. Although similar resistance mechanisms have not yet been reported in *S. aureus*, it is plausible that this species could adopt comparable strategies to evade QSI-mediated inhibition.

To minimize the potential of resistance driven by efflux activity, it may be advantageous to design QSIs that act extracellularly and do not require intracellular penetration [261]. Furthermore, mathematical models have been proposed to predict the emergence of resistance to QSIs [262], although these models require further experimental validation. In addition to genetic adaptations, bacteria can bypass quorum-sensing-dependent gene expression through epigenetic modifications, thereby reducing the efficacy of QSIs without altering the DNA sequence [263]. Resistance may also arise through other previously mentioned mechanisms, such as mutations in quorum-sensing genes or the compensatory upregulation of alternative regulatory systems [264].

### Quorum-sensing inhibitors translational challenges & clinical development

Despite extensive preclinical research and in vitro successes, the clinical development of QSIs faces numerous translational challenges. One of the primary limitations is the lack of well-defined clinical endpoints [265]. Unlike conventional antibiotics that exert direct bactericidal or bacteriostatic effects, QSIs function by attenuating bacterial virulence without reducing bacterial load, complicating the assessment of therapeutic efficacy using traditional indicators such as bacterial clearance or mortality reduction [31]. Moreover, regulatory agencies currently lack a standardized framework for evaluating antivirulence therapies, creating uncertainty in clinical trial design, efficacy endpoints, and approval pathways [266]. The success of QSIs often relies on the ability of the host immune system to eliminate less-virulent bacterial populations, raising concerns about their efficacy in immunocompromised patients [267].

Pharmacokinetic and pharmacodynamic limitations pose additional challenges [268]. Many QSI candidates exhibit limited stability, poor solubility, or low in vivo bioavailability, thereby limiting their ability to achieve effective concentrations at the site of infection without inducing toxicity [269]. Furthermore, some QSIs are also subject to rapid metabolic degradation and clearance, requiring structural modifications to enhance their half-life and systemic distribution [206]. Although several QSIs have demonstrated efficacy in animal models, few candidates have progressed to clinical trials owing to constraints in drug development. This lack of translational success has contributed to reduced pharmaceutical investment in the field.

Addressing these challenges will require not only the biological and chemical optimization of QSI candidates but also the development of antivirulence-specific clinical evaluation criteria, supportive regulatory frameworks, and innovative funding and investment models to incentivize advancement toward clinical application.

### Future perspectives

To address the aforementioned limitations, we propose a combinatorial therapeutic strategy that integrates QSIs and biofilm-disrupting agents to mitigate the adverse effects associated with QSIs monotherapy. Such a dual therapeutic approach can simultaneously inhibit biofilm formation and suppress virulence factor expression, thereby exerting synergistic effects to reduce bacterial virulence and mitigate AMR. Furthermore, integrating QSIs with conventional antibiotics could reduce the risk of resistance development by allowing reduced antibiotic doses and minimizing bacterial stress responses and adaptation [270, 271]. An emerging strategy in this context is to simultaneously target multiple signaling pathways to enhance the effectiveness of quorum-sensing inhibition and to make it more challenging for bacterial populations to coordinate virulence and establish persistent infections [272].

Innovative delivery systems should also be explored to enhance the clinical utility of QSIs. Nanoencapsulation technology, for instance, offers the potential for targeted and localized delivery of QSIs and biofilm-disrupting agents directly to infection sites [273]. This targeted delivery can minimize systemic exposure and associated off-target effects, while also protecting QSIs from premature degradation and facilitating sustained antimicrobial activity [274]. Additionally, advances in genomic sequencing and bioinformatics can help pinpoint active quorum-sensing circuits during bacterial infection, thereby facilitating tailored therapeutic interventions that selectively inhibit pathogenic signaling pathways [275, 276]. Furthermore, emerging machine learning approaches could enhance treatment optimization by predicting bacterial resistance profiles and identifying effective drug combinations [277].

Emerging technologies, such as microfluidic platforms and lab-on-a-chip devices, are being developed to enable real-time monitoring of bacterial quorum-sensing activity and biofilm dynamics in patient-derived specimens [278]. Such advances could empower clinicians to make timely, data-driven treatment decisions tailored to individual infections. However, further research is still required to establish clinical guidelines and validate the efficacy and safety of personalized QSI-based therapies in human populations.

## Conclusion

Quorum-sensing systems are key regulators of bacterial pathogenicity. They orchestrate the expression of virulence factors and facilitate colonization once bacterial populations have reached a critical density sufficient to withstand environmental and host-induced stressors. In *S. aureus*, the agr system serves as the primary quorum-sensing mechanism and functions as a global regulator for multiple virulence factors. QSIs disrupt these communication networks, effectively reducing the expression of virulence determinants and thereby rendering bacterial cells more susceptible to antimicrobial therapies. QSIs can exert their effects through multiple mechanisms: obstructing the synthesis of AIPs, degrading existing AIPs, competitively inhibiting and modifying quorum-sensing receptors, or interfering with regulatory components of quorum-sensing pathways.

Biofilms are a key virulence factor in *S. aureus*, partially regulated by quorum sensing during the later stages of development, thereby contributing to biofilm dispersal and virulence factor expression. While QSIs alone cannot be considered as antibiofilm agents, their use, particularly during the post-exponential phases, may inadvertently promote biofilm formation. Consequently, treating chronic infections requires a multifaceted therapeutic approach that combines agents targeting virulence regulation with those capable of disrupting established biofilms. Such a strategy could overcome one of the significant limitations of QSIs therapy: the persistence of biofilms due to blockade of the agr system, which plays a pivotal role in biofilm dispersal.

Targeting bacterial virulence factors, including those regulated by quorum sensing, offers a compelling therapeutic approach with diverse applications. Such strategies hold promise for preventing device-associated infections, enhancing bacterial susceptibility to conventional antibiotics, and managing wound environments. However, further research is essential to translate these strategies into effective clinical interventions, particularly for persistent, biofilm-associated infections.

## Data Availability

No datasets were generated or analysed during the current study.

## Abbreviations

AHLs: Acyl-homoserine lactones
Agr: Accessory gene regulator
AIP: Autoinducing peptide
AMR: Antimicrobial resistance
Coa: Coagulase
CWA: Cell wall-anchored proteins
eDNA: Extracellular DNA
ECM: Extracellular matrix
EPS: Exopolysaccharides
Hla: Alpha-hemolysin
MRSA: Methicillin-resistant Staphylococcus aureus
MSCRAMMs: Microbial surface components recognizing adhesive matrix molecules
PBP: Penicillin-binding protein
PIA: Polysaccharide intercellular adhesin
PSMs: Phenol-soluble modulins
QSIs: Quorum-sensing inhibitors
ROT: Repressor of toxins
Sar: Staphylococcal accessory regulator
TCSs: Two-component systems
